# Targeted Quantitative Polymerase Chain Reaction Analysis of Gut Microbiota and Its Association With Feeding and Mealtime Behavior and Intestinal Permeability in Children With Autism Spectrum Disorder

**DOI:** 10.14740/jocmr6687

**Published:** 2026-10-23

**Authors:** Walaa H. Ali, Abdulmabod Omar, Amal H. Ali, Hesham M. Hefny, Gehad Nabil, Yasmin Mahmoud, Asmaa Elsaudi Abdelgawad Nasr, Salwa M. Abdelhamid, Hala Mohammed Amin, Marwa Mohamed Omar, Lama M. El-Attar, Fatma Saffeyeldin Mohamed, Rasha Elgamal, Mohamed Ibrahem, Hala S.M. Abdelmotogaly

**Affiliations:** a Child Health Department, National Research Centre, Dokki, Giza, Egypt; b Department of Clinical Pathology, Faculty of Medicine, Al-Azhar University, Cairo, Egypt; c Department of Medical Microbiology and Immunology, Faculty of Medicine, Aswan University, Aswan, Egypt; d Clinical Pathology Department, Faculty of Medicine, Sohag University, Sohag, Egypt; e Department of Pediatrics, Faculty of Medicine for Girls, Al-Azhar University, Cairo, Egypt; f Department of Neuropsychiatry, Okasha Institute of Psychiatry, Faculty of Medicine, Ain Shams University, Cairo, Egypt; g Department of Clinical Pathology, Faculty of Medicine for Girls, Al-Azhar University, Cairo, Egypt; h Department of Pediatrics, Faculty of Medicine, Zagazig University, Zagazig, Egypt; i Department of Clinical Pathology, Faculty of Medicine, Menoufia University, Shebin El-Kom, Egypt; j Department of Human Genetics, Medical Research Institute, Alexandria University, Alexandria, Egypt; k Department of Hepatogastroenterology and Infectious Diseases, Faculty of Medicine for Girls, Al-Azhar University, Cairo, Egypt; l Department of Internal Medicine, College of Medicine, Taif University, Taif, Saudi Arabia; m Department of Clinical Pathology, Faculty of Medicine, Suez University, Suez, Egypt; n Department of Medical Biochemistry and Molecular Biology, Faculty of Medicine, Cairo University, Cairo, Egypt; o Department of Pediatrics and Neonatology, Faculty of Medicine, New Valley University, New Valley, Egypt

**Keywords:** Autism spectrum disorder, Gut microbiome, Feeding and mealtime behavior, Intestinal permeability, Zonulin, Calprotectin, Dysbiosis

## Abstract

**Background:**

Gastrointestinal (GI) symptoms and feeding and mealtime problems are common in children with autism spectrum disorder (ASD). It is important to note that the gut microbiome is a hypothesized mechanism of action, but it is not yet known if specific bacterial profiles and markers of intestinal barrier function predict feeding problems in the target group of ASD.

**Methods:**

This is a cross-sectional case–control study that examined 70 children with ASD (aged 3–12 years) and 70 matched neurotypical children. Autism was evaluated using the Childhood Autism Rating Scale (CARS), and mealtime behaviors were evaluated using the Brief Autism Mealtime Behavior Inventory (BAMBI). Six important bacterial taxa were quantified using real-time polymerase chain reaction (PCR), and fecal zonulin and calprotectin were quantified by enzyme-linked immunosorbent assay (ELISA).

**Results:**

Children with ASD had significantly higher levels of fecal zonulin and calprotectin than controls (both at P < 0.001) and lower abundances of beneficial taxa (*Prevotella*, *Bifidobacterium*, *Lactobacillus*) and higher abundances of *Clostridium difficile* and *Bacteroidetes* (all P < 0.01). The overall BAMBI scores did not differ among the groups, but within the ASD group, feeding problem severity was highly correlated with lower *Bifidobacterium* (r = −0.68) and higher *Clostridium difficile* (r = 0.74) and was not significantly correlated with autism severity, zonulin, or calprotectin.

**Conclusion:**

Children with ASD have increased levels of gut dysbiosis and barrier dysfunction compared to their non-autistic peers, but the severity of their feeding and mealtime problems tracked the degree of gut microbial imbalance rather than the severity of their ASD symptoms in general. This suggests that feeding behavior is a characteristic that has been under-recognized as a component of the ASD phenotype and is related to the microbiome; if confirmed in longitudinal studies, these microbial associations could represent a candidate target for future gut-directed intervention research.

## Introduction

Persistent deficits in social communication and restricted, repetitive patterns of behavior characterize autism spectrum disorder (ASD), which is a neurodevelopmental condition [1]. It has increased over the last 20 years and is now defined as a significant public health problem affecting children [2]. In addition to the basic behaviors, children with ASD are more likely to have gastrointestinal (GI) problems and feeding issues such as food refusal, highly selective eating, and rigid feeding patterns that are sensory driven [3, 4]. These feeding issues are no trivial matter, with reports of micronutrient deficiencies, poor growth, and even exacerbation of behavioral symptoms [5, 6].

More research has emerged indicating that the gut is not just a spectator in these scenes. The gut–brain axis is a bidirectional communication system linking the intestinal microbiota to the central nervous system via neural, immune, and endocrine pathways, which has been suggested to be a mechanistic link between intestinal dysfunction and neurobehavioral symptoms in ASD [7, 8]. The effects of microbial metabolites on neurodevelopment and behavior are yet to be fully understood and attributed to the integrity of the gut barrier [9, 10]. Two biomarkers have been found to be useful for investigating this barrier non-invasively: fecal zonulin, a marker of the integrity of tight junctions and paracellular permeability, and fecal calprotectin, a marker of neutrophil-dependent mucosal inflammation [11, 12]. It is already known that children with ASD have elevated zonulin, which is consistent with a heritable component in gut barrier dysfunction, and that decreased *Prevotella* has been repeatedly reported as one of the more consistent microbial signatures of ASD [13, 14].

Multiple independent groups have reported changes to the gut microbial community in ASD, although the taxa that are implicated differ somewhat across studies [15, 16], and gut microbial signals are consistently correlated with GI and behavioral severity in animal and human studies alike [17]. Short courses of oral vancomycin or longer-term microbiota transfer therapy have resulted in transient improvements in GI and core ASD symptoms with at least suggestive causal, not correlational, links [18, 19].

Most of this literature has been limited to an examination of either gut microbial composition or intestinal permeability and inflammation and/or feeding specific behavior, but not both in the same sample of pediatric ASD. This study was developed to fill that void. We explored the composition of gut microbiota, fecal zonulin, and fecal calprotectin in children with ASD and in neurotypical children, and in the ASD group, we investigated their relationship with the severity of autism as well as with mealtime and feeding behaviors.

## Materials and Methods

### Study design and setting

This study was conducted as a cross-sectional, analytical case-control study at the outpatient clinics at Al-Azhar University Hospitals, Egypt, following the ethical principles established by the Declaration of Helsinki [20], and ethical approval was obtained from the Institutional Review Board of Al-Azhar University Hospitals prior to enrolment (IRB Number: Clin-Path-R-2026-7-001). Written informed consent was obtained from a parent or legal guardian of every participating child, and all patient data were handled confidentially and stored under restricted access, in keeping with Helsinki’s requirements for the protection of research participants and their personal data.

### Study population

The study enrolled 70 children aged 3–12 years with a confirmed diagnosis of ASD (case group) and 70 age- and sex-matched neurotypical children recruited from general pediatric clinics (control group). ASD diagnosis was established according to the Diagnostic and Statistical Manual of Mental Disorders (DSM-5) criteria [1] and confirmed with the Autism Diagnostic Observation Schedule, Second Edition (ADOS-2) [21]. Controls had no ASD diagnosis and no major neuropsychiatric disorder. Children with inflammatory bowel disease, chronic liver disease, autoimmune disease, a known genetic syndrome, immunosuppressive therapy, or recent (< 4 weeks) antibiotic or probiotic use were excluded from either group, since each of these can independently alter gut permeability or microbial composition.

Controls were matched to the ASD group at the group level (frequency matching) on age and sex, such that the aggregate age and sex distributions of the two groups were comparable (Table 1), rather than through individual case-by-case pairing. No a priori sample-size calculation was performed for this study; given the very large between-group effect sizes observed for the primary fecal zonulin and calprotectin comparisons (Cohen’s d > 2.5; Table 2), the achieved sample of 70 children per group provided in excess of 99% post-hoc power to detect these differences at α = 0.05, although the study may have been comparatively under-powered for smaller, taxon-specific abundance comparisons restricted to detection-positive subsamples (Table 3).

**Table 1:** Demographic and Perinatal Characteristics of the Study Groups

Variable	ASD (N = 70)	Control (N = 70)	Test statistic	P value
Age, years, M (SD)	8.2 (2.3)	7.9 (2.2)	t = 0.72	0.472
Sex, n (%)				
Male	27 (38.6)	29 (41.4)	χ^2^ = 0.03	0.863
Female	43 (61.4)	41 (58.6)		
Residence, n (%)				
Urban	37 (52.9)	38 (54.3)	χ^2^ = 0.00	1.000
Rural	33 (47.1)	32 (45.7)		
Family income, n (%)				
Enough	25 (35.7)	21 (30.0)	χ^2^ = 0.86	0.650
Enough and saving	23 (32.9)	28 (40.0)		
Not enough	22 (31.4)	21 (30.0)		
Father’s education, n (%)				
Basic/none	23 (32.9)	28 (40.0)	χ^2^ = 2.37	0.306
Primary/secondary	30 (42.9)	32 (45.7)		
University	17 (24.3)	10 (14.3)		
Mother’s education, n (%)				
Basic/none	27 (38.6)	28 (40.0)	χ^2^ = 1.06	0.589
Primary/secondary	32 (45.7)	27 (38.6)		
University	11 (15.7)	15 (21.4)		
Parental smoking, n (%)				
Yes	40 (57.1)	35 (50.0)	χ^2^ = 0.46	0.498
No	30 (42.9)	35 (50.0)		
Siblings, n, M (SD)	1.7 (1.5)	1.7 (1.4)	t = 0.00	1.000
Low birth weight, n (%)				
Yes	43 (61.4)	30 (42.9)	χ^2^ = 4.12	0.042
No	27 (38.6)	40 (57.1)		
Full-term delivery, n (%)				
Yes	38 (54.3)	47 (67.1)	χ^2^ = 1.92	0.166
No	32 (45.7)	23 (32.9)		
NICU admission, n (%)				
Yes	37 (52.9)	38 (54.3)	χ^2^ = 0.00	1.000
No	33 (47.1)	32 (45.7)		
Weight-for-age z-score, M (SD)	0.4 (2.0)	−0.1 (1.9)	t = 1.53	0.129
Height-for-age z-score, M (SD)	0.1 (2.0)	0.1 (2.1)	t = −0.01	0.993

Percentages are within-group column percentages and may not sum to 100 due to rounding. M: mean; SD: standard deviation; NICU: neonatal intensive care unit; ASD: autism spectrum disorder.

**Table 2:** Fecal Biomarkers of Intestinal Permeability and Inflammation

Biomarker	ASD (N = 70)	Control (N = 70)	Test statistic (t)	P value
Fecal zonulin, ng/mL (mean ± SD)	133.5 ± 25.6	59.8 ± 17.5	t = 19.84	< 0.001
Fecal calprotectin, µg/g (mean ± SD)	151.1 ± 42.2	61.8 ± 24.1	t = 15.39	< 0.001

SD: standard deviation; ASD: autism spectrum disorder.

**Table 3:** Gut Bacterial Taxa: Detection Rate and Abundance (Log_10_ CFU/g) in ASD versus Control Groups

Taxon	Detected, ASD N (%)	Detected, control N (%)	Detection χ^2^, P	Abundance*, Log_10_ CFU/g (ASD vs. control), t, P
*Prevotella*	38 (54.3)	65 (92.9)	χ^2^ = 24.83, P < 0.001	5.5 ± 0.8 vs. 7.8 ± 0.7; t = −14.35, P < 0.001
*Bifidobacterium*	32 (45.7)	59 (84.3)	χ^2^ = 21.22, P < 0.001	5.4 ± 0.8 vs. 7.7 ± 0.8; t = −14.00, P < 0.001
*Lactobacillus*	30 (42.9)	68 (97.1)	χ^2^ = 46.56, P < 0.001	5.7 ± 0.7 vs. 7.7 ± 0.7; t = −12.49, P < 0.001
*Clostridium difficile*	51 (72.9)	34 (48.6)	χ^2^ = 7.67, P = 0.006	7.5 ± 0.9 vs. 4.0 ± 0.6; t = 21.10, P < 0.001
*Bacteroidetes*	36 (51.4)	26 (37.1)	χ^2^ = 2.34, P = 0.126	7.4 ± 1.0 vs. 4.1 ± 0.7; t = 15.72, P < 0.001
*Firmicutes*	38 (54.3)	53 (75.7)	χ^2^ = 6.15, P = 0.013	7.5 ± 1.1 vs. 4.3 ± 0.6; t = 16.30, P < 0.001

*Among taxon-positive samples only (n varies by taxon; see Section “Gut microbiota composition” text and Methods).

### Data collection

Demographic and perinatal data (age, sex, residence, family income, parental education and occupation, parental smoking, number of siblings, birth weight, gestational term, and neonatal intensive care unit (NICU) admission) were obtained from caregivers through structured interviews. Weight and height were measured with calibrated equipment and converted to weight-for-age (WAZ) and height-for-age (HAZ) z-scores using the World Health Organization (WHO) reference standards. Autism severity in the case group was rated with the Childhood Autism Rating Scale (CARS), a 15-item clinician-administered instrument in which higher totals indicate more severe autistic symptomatology [22]. Mealtime and feeding behavior were assessed in both groups with the Arabic version of the Brief Autism Mealtime Behavior Inventory (BAMBI), an 18-item caregiver-reported questionnaire covering food refusal, limited food variety, and autism-specific mealtime behaviors, each rated from 1 (never/rarely) to 5 (almost always) [23].

Severity subgroup (mild/moderate/severe, Section “Autism severity and mealtime/feeding behavior”) was assigned by the evaluating clinician as part of the overall diagnostic impression, incorporating history and clinical observation alongside the CARS, rather than derived solely from a fixed CARS total-score cut-off; readers should not assume a direct score-based correspondence between the CARS total reported here and the severity subgroup label. All ADOS-2 assessments were conducted by clinicians trained and certified in ADOS-2 administration, using the module (Toddler, 1, 2, or 3) appropriate to each child’s age and expressive language level, according to standard ADOS-2 administration guidelines.

The BAMBI is a caregiver-report screening measure of mealtime behavior and food selectivity; it is not a diagnostic instrument, and no formal diagnostic assessment for feeding or eating disorders (e.g., avoidant/restrictive food intake disorder, anorexia nervosa, or bulimia nervosa) was performed in either group in this study. Throughout this manuscript we therefore refer to “feeding and mealtime problems” rather than “eating disorders.” The BAMBI was originally developed and validated in a sample that included both children with autism and typically developing children [23], supporting its use as a caregiver-report measure in both groups in the present study.

#### Gut microbiota analysis

Fresh stool samples were collected by the participants’ legal guardians at home using sterile collection containers. Samples were transported to the laboratory within 2 h of collection, aliquoted upon arrival, and stored at −80 °C until analysis. Microbial DNA was extracted from approximately 200 mg of each fecal sample using the SKYAMP STOOL DNA Kit (Art. No. EDC328, SkyGen, Beijing, China) according to the manufacturer’s instructions. DNA concentration and purity were assessed spectrophotometrically before molecular analysis. Quantitative real-time polymerase chain reaction (qPCR) was performed to determine the abundance of six bacterial taxa selected based on their reported association with ASD and gut microbial dysbiosis: *Prevotella*, *Bifidobacterium*, *Lactobacillus*, *Clostridium difficile*, *Bacteroidetes*, and *Firmicutes*. Taxon-specific primers targeting the bacterial 16S rRNA gene were used for amplification. Reactions were carried out using SYBR Green chemistry in a real-time PCR system according to the manufacturer’s recommendations. Each reaction included appropriate negative controls and positive reference samples. Amplification specificity was confirmed by the melting-curve analysis. Samples were considered positive when amplification occurred within the predefined threshold cycle (Ct). For each bacterial taxon, both the detection frequency (number and percentage of positive samples) and the quantitative bacterial abundance among positive samples were recorded and compared between the ASD and control groups.

Bacterial loads are reported as Log_10_ colony-forming units (CFU)-equivalents per gram of stool, back-calculated from qPCR cycle threshold values against a standard curve of known template copy number and an assumed average genome-copy-per-cell value; this qPCR-derived quantity is a relative, estimated equivalent rather than a value calibrated against culture-based colony counts, and should be interpreted as a semi-quantitative index of taxon abundance rather than an absolute bacterial count. Taxon-specific primer pairs (5’–3’), adopted from Nouh et al [24], were *Prevotella*—PrevF CACCAAGGCGACGATCA/PrevR GGATAACGCCYGGACCT; *Bifidobacterium*—Bif-F TCGCGTC(C/T)GGTGTGAAAG/Bif-R CCACATCCAGC(A/G)TCCAC; *Lactobacillus*—Lacto-F AGCAGTAGGGAATCTTCCA/Lacto-R CACCGCTACACATGGAG; *Bacteroidetes*—Bact934F GGARCATGTGGTTTATTCGATGAT/Bact1060R AGCTGACGACAACCATGCAG; and *Firmicutes*—Firm934F GGAGYATGTGGTTTAATTCGAAGCA/Firm1060R AGCTGACGACAACCATGCAC. Reactions were run in a 20-µL volume containing 4 pmol of each primer, with thermal cycling of initial denaturation at 95 °C for 10 min, followed by 40 cycles of denaturation at 95 °C for 30 s, annealing at 60 °C for 30 s, and extension at 72 °C for 30 s, on a Rotor-Gene Q cycler (Qiagen, Germany) using a SensiFAST SYBR No-ROX PCR kit (Bioline Co., UK) [24]. The Clostridium primers used were species-specific for *Clostridium difficile* (*C. diff*-F TTGAGCGATTTACTTCGGTAAAGA/*C. diff*-R TGTACTGGCTCACCTTTGATATTCA); accordingly, this taxon is reported as *Clostridium difficile*, rather than at the genus level, throughout this manuscript’s text, tables, and figures. Note that Nouh et al [24], the source of these primers, themselves quantified taxa as relative abundance normalized against total bacterial DNA (universal 16S rRNA) rather than as Log_10_ CFU-equivalents; the Log_10_ CFU-equivalent quantification reported throughout this manuscript was generated independently for this study using a standard curve of known template copy number, as described above, rather than by adopting the source publication’s relative-abundance approach. These primer sets were originally designed and validated by Rinttila et al [25], who reported a linear range of quantification for this assay family from 0.1–10 pg to 10 ng of target genomic DNA, corresponding to approximately from 30–4,500 to 1.9 × 10^6^–6.0 × 10^6^ target bacterial genomes. The amplification efficiency (%) and R^2^ of the standard curve generated for this study, the Ct cut-off used to define a positive sample, and batch/plate information were not retained in a form suitable for reporting here; readers seeking this instrument-level detail may contact the corresponding author. We have verified the primer sequences above directly against the original source document. Because normalization to total bacterial load was not performed, the abundance values in Table 3 should be interpreted as comparisons of taxon-specific Log_10_ CFU-equivalents rather than of the taxon’s proportional representation within the community.

#### Fecal zonulin (intestinal permeability)

Fecal zonulin concentrations were determined using a competitive enzyme-linked immunosorbent assay (ELISA) (IDK Zonulin ELISA, Immundiagnostik AG, Bensheim, Germany; REF K5600) according to the manufacturer’s protocol in both the ASD and control groups. Stool extracts were incubated with a biotinylated zonulin tracer in microplate wells coated with polyclonal anti-zonulin antibodies. Free zonulin in the samples competed with the tracer for antibody binding. After washing, peroxidase-conjugated streptavidin and tetramethylbenzidine (TMB) substrate were added. The reaction was terminated with an acidic stop solution, and absorbance was measured spectrophotometrically. According to the manufacturer, the median fecal zonulin concentration in healthy individuals was 61 ± 46 ng/mL. Zonulin concentrations were classified as normal (< 83.15 ng/mL), elevated (83.15–110.0 ng/mL), or high (> 110.0 ng/mL).

#### Fecal calprotectin (intestinal inflammation)

Fecal calprotectin concentrations were determined using a sandwich ELISA (Calprotectin ELISA, BUHLMANN Laboratories AG, Schonenbuch, Switzerland; REF EK-CAL) according to the manufacturer’s instructions. Stool extracts were added to microplate wells coated with monoclonal anti-calprotectin antibodies. Bound calprotectin was detected using an enzyme-labeled secondary antibody, followed by substrate development and spectrophotometric measurement of absorbance. According to the manufacturer, fecal calprotectin concentrations were classified as normal (< 80 µg/g), borderline (80–160 µg/g), or elevated (> 160 µg/g).

### Outcome definitions

The primary outcome was the association between gut microbial composition/intestinal biomarkers and feeding/mealtime problem severity, operationalized as the Total BAMBI score and its three subscales (food refusal, limited food variety, and autism-specific mealtime behavior) within the ASD group. Secondary outcomes were between-group differences in fecal zonulin, fecal calprotectin, and taxon detection rate/abundance, and the correlation of these measures with autism severity (CARS score) within the ASD group.

### Statistical analysis

Data were analyzed using IBM SPSS Statistics, version 26.0. Continuous variables are presented as mean ± standard deviation (SD) and were compared between groups using the independent-samples *t*-test (Welch’s correction where variances were unequal). Categorical variables are presented as number (%) and were compared using the chi-square test. Associations between continuous clinical, biomarker, and microbiota variables within the ASD group were examined using Spearman’s rank correlation coefficient (r), given the skewed distribution of several variables. A multiple linear regression was used to explore joint predictors of Total BAMBI score among cases with complete taxon data. A two-tailed P value < 0.05 was considered statistically significant throughout.

## Results

### Characteristics of participants

A total of 140 children were enrolled: 70 with ASD and 70 neurotypical controls. The two groups were well matched; there were no significant differences in age, sex distribution, residence, family income, parental education, parental smoking, number of siblings, WAZ, or HAZ (Table 1). Low birth weight was more frequent in the ASD group (61.4% vs. 42.9%, P = 0.042); full-term delivery and NICU admission did not differ significantly between groups.

### Autism severity and mealtime/feeding behavior

Within the ASD group, the mean CARS score was 39.5 ± 5.7 (range: 30.1–47.8), with 28 children (40.0%) classified as mild, 22 (31.4%) as moderate, and 20 (28.6%) as severe. Mean age at diagnosis was 4.0 ± 1.2 years. Total BAMBI scores did not differ significantly between children with ASD and controls, and none of the three BAMBI subscales showed a significant between-group difference (Table 4); both groups reported a comparable overall burden of problematic mealtime behavior on this instrument.

**Table 4:** Autism Severity and Mealtime/Feeding Behavior (BAMBI) by Group

Variable	ASD (N = 70)	Control (N = 70)	Test statistic (t)	P value
CARS total score (mean ± SD)	39.5 ± 5.7			
Age at diagnosis, years (mean ± SD)	4.0 ± 1.2			
Total BAMBI score (mean ± SD)	54.1 ± 6.3	54.7 ± 5.5	t = −0.60	0.547
Food refusal subscale (mean ± SD)	15.1 ± 3.6	15.4 ± 3.1	t = −0.57	0.566
Limited food variety subscale (mean ± SD)	23.6 ± 4.1	23.9 ± 3.7	t = −0.33	0.745
Autism-specific mealtime behavior subscale (mean ± SD)	15.4 ± 3.5	15.5 ± 2.8	t = −0.11	0.915

CARS: Childhood Autism Rating Scale; BAMBI: Brief Autism Mealtime Behavior Inventory; SD: standard deviation; ASD: autism spectrum disorder.

### Fecal biomarkers of intestinal permeability and inflammation

Children with ASD had substantially higher fecal zonulin and calprotectin concentrations than controls (Table 2, Fig. 1). The mean fecal zonulin concentration in the ASD group (133.5 ng/mL) fell within the manufacturer’s “high” category (> 110.0 ng/mL), consistent with increased intestinal permeability; however, the mean fecal calprotectin concentration in the ASD group (151.1 µg/g) fell within the manufacturer’s “borderline” category (80–160 µg/g) rather than the “elevated” category (> 160 µg/g); therefore, these findings should be read as indicating a statistically robust but, on average, only borderline elevation in mucosal inflammatory marker levels rather than clearly elevated inflammation in most children with ASD. Zonulin and calprotectin were not significantly correlated with one another within the ASD group (r = 0.17, P = 0.166), suggesting the two processes, while both elevated, are not simply tracking the same underlying signal in this sample.

**Figure 1:**
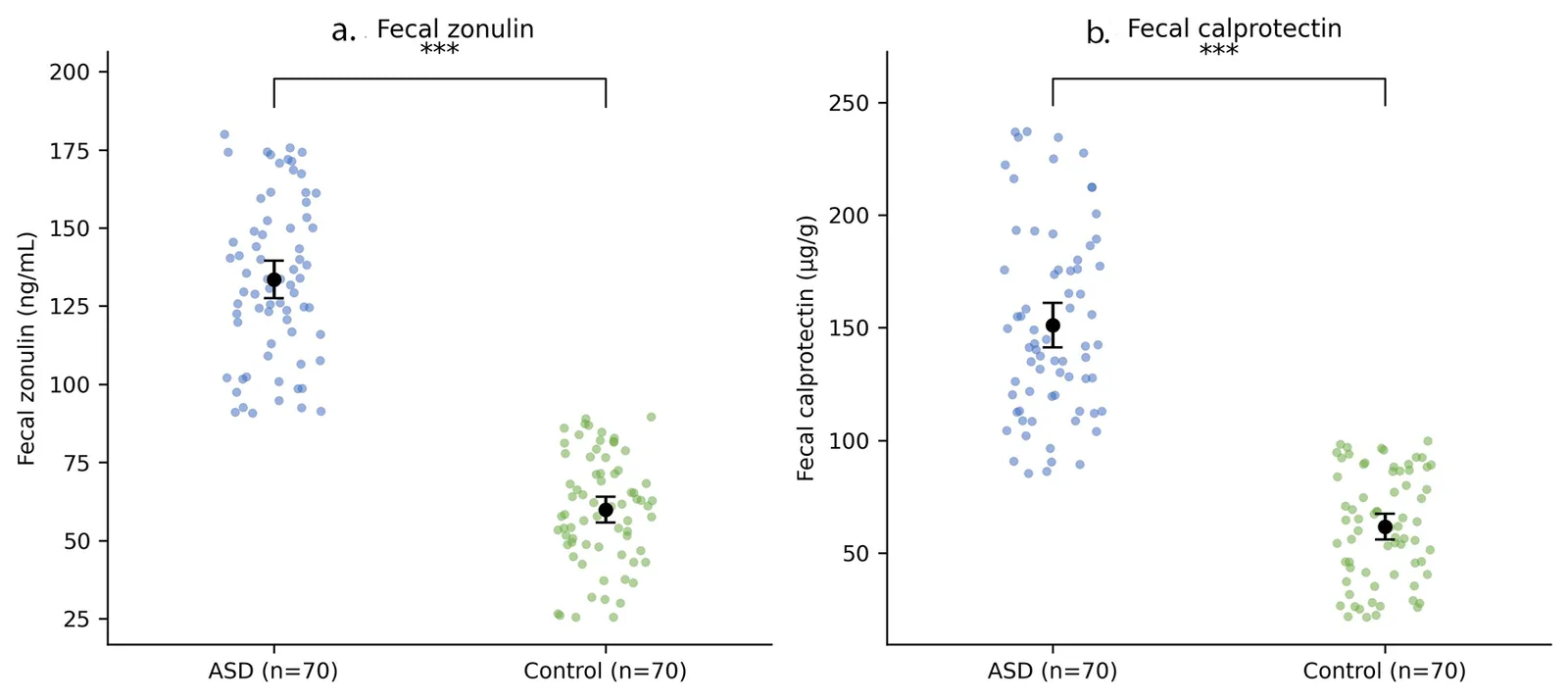
Fecal zonulin (a) and fecal calprotectin (b) concentrations in children with ASD versus neurotypical controls (mean ± SD; ***P < 0.001). ASD: autism spectrum disorder; SD: standard deviation.

Effect sizes for the two group comparisons in Table 2 (Cohen’s d, with 95% confidence interval (CI), calculated from the reported *t*-statistics and n = 70 per group) were very large: fecal zonulin d = 3.35 (95% CI: 2.84–3.86) and fecal calprotectin d = 2.60 (95% CI: 2.15–3.05). It should also be noted that fecal zonulin is not yet a standardized clinical biomarker; assay performance, cut-off values, and reference ranges vary across commercial kits and populations, and the zonulin findings reported here should therefore be interpreted as one operationalization of intestinal permeability rather than a definitive clinical measure.

### Gut microbiota composition

Targeted qPCR quantification showed a consistent pattern of dysbiosis in the ASD group (Table 3). *Prevotella*, *Bifidobacterium*, and *Lactobacillus* were detected less frequently in children with ASD than in controls, and, among samples in which they were detected, their abundance was also significantly lower. Conversely, *Clostridium difficile* was both more frequently detected and more abundant in ASD, and *Bacteroidetes*, while detected at a similar rate in the two groups, reached markedly higher abundance among ASD-positive samples. *Firmicutes* showed a mixed pattern: it was detected less often in ASD (54.3% vs. 75.7% of samples) but, when present, occurred at nearly twice the abundance seen in controls.

The comparatively low detection rates for *Prevotella, Bifidobacterium,* and *Lactobacillus* in the ASD group (42.9–54.3% of samples) are interpreted here as reflecting genuine depletion of these taxa below the assay’s limit of detection in a substantial subset of children with ASD, consistent with prior reports of near-absence (rather than simple under-abundance) of these genera in autism; however, we cannot fully exclude technical non-detection, and this is addressed further in the Limitations. Abundance (Log_10_ CFU/g) was compared only among taxon-positive samples because assigning an arbitrary numeric value to non-detected samples (e.g., zero or an assumed limit-of-detection value) would have biased the abundance comparison in an unpredictable direction; this approach is explicitly a limitation, as it restricts the abundance comparisons in Table 3 to the subset of children in whom each taxon was detectable, and detection status itself (rather than abundance) is the more complete comparison for taxa with low detection rates. Applying a Bonferroni correction for the six detection-rate comparisons in Table 3 (α = 0.05/6 = 0.0083), the between-group differences in detection rate remained significant for *Prevotella, Bifidobacterium, Lactobacillus,* and *Clostridium difficile*, but the difference in *Firmicutes* detection rate (54.3% vs. 75.7%, uncorrected P = 0.013) did not survive correction and should be regarded as exploratory. All six abundance comparisons remained significant after the same correction (all corrected P < 0.006). Effect sizes for the abundance comparisons were uniformly very large (Cohen’s d, with 95% CI, calculated from the reported *t*-statistics and taxon-positive sample sizes): *Prevotella* d = −2.93 (95% CI: −3.50 to −2.36), *Bifidobacterium* d = −3.07 (95% CI: −3.69 to −2.45), *Lactobacillus* d = −2.74 (95% CI: −3.32 to −2.16), *Clostridium difficile* d = 4.67 (95% CI: 3.85–5.50), *Bacteroidetes* d = 4.05 (95% CI: 3.18–4.92), and *Firmicutes* d = 3.47 (95% CI: 2.82–4.12). We note that effect sizes of this magnitude are considerably larger than is typical in the microbiome-ASD literature and most likely reflect the narrow within-group variability of the Log_10_-transformed qPCR values in this cohort; independent replication would strengthen confidence in the absolute magnitude of these differences.

### Correlations between feeding/mealtime problem severity, autism severity, biomarkers, and microbiota

Within the ASD group, neither CARS score nor Total BAMBI score correlated significantly with fecal zonulin, fecal calprotectin, or with each other (Table 5). However, Total BAMBI score correlated significantly with the abundance of every taxon examined except *Firmicutes*: negatively with *Prevotella*, *Bifidobacterium*, and *Lactobacillus*, and positively with *Clostridium difficile* and *Bacteroidetes* (Table 5, Fig. 2). None of the six taxa correlated significantly with the CARS score. In other words, the severity of feeding and mealtime problems tracked the child’s gut bacterial profile far more closely than it tracked overall autism severity or systemic inflammatory/permeability markers.

**Table 5:** Spearman’s Correlations Within the ASD Group (N = 70, or as Noted)

Variables compared	Spearman's r	P value	N
CARS vs. fecal zonulin	0.03	0.815	70
CARS vs. fecal calprotectin	−0.06	0.618	70
Total BAMBI vs. fecal zonulin	−0.04	0.754	70
Total BAMBI vs. fecal calprotectin	0.03	0.806	70
CARS vs. total BAMBI	−0.04	0.724	70
Total BAMBI vs. *Prevotella* abundance	−0.35	0.029	38
Total BAMBI vs. *Bifidobacterium* abundance	−0.68	< 0.001	32
Total BAMBI vs. *Lactobacillus* abundance	−0.41	0.023	30
Total BAMBI vs. *Clostridium difficile* abundance	0.74	< 0.001	51
Total BAMBI vs. *Bacteroidetes* abundance	0.63	< 0.001	36
Total BAMBI vs. *Firmicutes* abundance	0.20	0.237	38
CARS vs. any of the six taxa	−0.10 to 0.23	All > 0.16	30–51

ASD: autism spectrum disorder; CARS: Childhood Autism Rating Scale; BAMBI: Brief Autism Mealtime Behavior Inventory.

**Figure 2:**
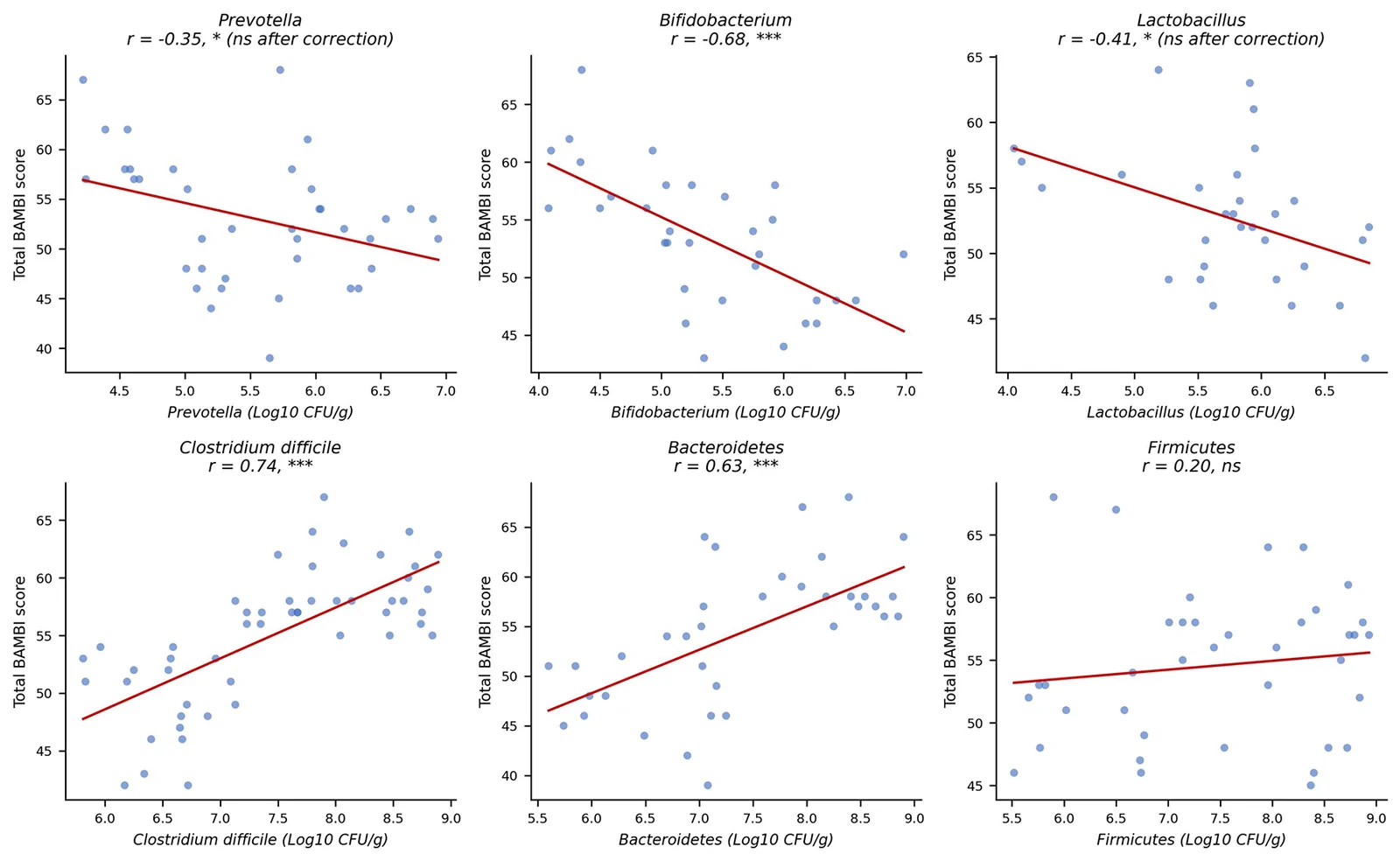
Scatterplots of Total BAMBI score against gut bacterial taxon abundance (Log_10_ CFU/g) within the ASD group, one panel per taxon, each showing individual children’s data. points and a linear trend line. Spearman’s r and significance are reported per panel (*P < 0.05, **P < 0.01, ***P < 0.001, ns: not significant); correlations that were significant at the uncorrected α = 0.05 level but did not survive Bonferroni correction for the 11 correlations tested (α = 0.0045) are labeled accordingly. ASD: autism spectrum disorder; BAMBI: Brief Autism Mealtime Behavior Inventory; CFU: colony-forming units.

The correlations above are reported at the conventional uncorrected α = 0.05 threshold. Applying a Bonferroni correction for the 11 correlations tested within the ASD group in Table 5 (α = 0.05/11 = 0.0045), the *Bifidobacterium* (r = −0.68, 95% CI: −0.83 to −0.43), *Clostridium difficile* (r = 0.74, 95% CI: 0.58–0.84), and *Bacteroidetes* (r = 0.63, 95% CI: 0.38–0.79) correlations with Total BAMBI score remained significant, whereas the *Prevotella* (r = −0.35, 95% CI: −0.60 to −0.03) and *Lactobacillus* (r = −0.41, 95% CI: −0.67 to −0.06) correlations did not survive correction and should be regarded as nominal or trend-level associations rather than confirmed findings. The *Firmicutes* correlation (r = 0.20, 95% CI: −0.13 to 0.49) remained non-significant under either approach. We report both the uncorrected and Bonferroni-corrected results transparently and, in the Discussion, restrict our strongest interpretive claims to the three taxa that survived correction.

### Joint predictors of feeding/mealtime problem severity (exploratory)

Because CARS, fecal zonulin, and fecal calprotectin showed no meaningful association with BAMBI score, a multiple linear regression including these three variables plus age explained essentially none of the variance in Total BAMBI (R^2^ = 0.004, P = 0.991) and is not reported in tabular form. In the smaller subsample of children with both *Bifidobacterium* and *Clostridium difficile* quantifiable (n = 22), a two-predictor linear regression explained just over half of the variance in Total BAMBI score (R^2^ = 0.584, P < 0.001; Table 6), with higher *Clostridium difficile* and lower *Bifidobacterium* abundance each independently associated with more severe feeding problems. Given the small subsample, this model should be read as hypothesis-generating rather than confirmatory.

**Table 6:** Multiple Linear Regression Predicting Total BAMBI Score From *Bifidobacterium* and *Clostridium difficile* Abundance (N = 22)

Predictor	β (unstandardized)	t	P value
Constant	44.09	4.06	0.001
*Bifidobacterium*, Log_10_ CFU/g	−2.67	−2.38	0.028
*Clostridium difficile*, Log_10_ CFU/g	3.32	3.36	0.003

Model R^2^ = 0.584, adjusted R^2^ = 0.540, F(2, 19) = 13.35, P < 0.001. CFU: colony-forming units.

Ninety-five percent CIs for the regression coefficients in Table 6, calculated from the reported unstandardized β and *t*-statistics (df = 19), were constant 44.09 (95% CI: 21.35–66.83), *Bifidobacterium* −2.67 (95% CI: −5.02 to −0.32), and *Clostridium difficile* 3.32 (95% CI: 1.25–5.39). With only n = 22 and two predictors, formal residual diagnostics (linearity, homoscedasticity, normality of residuals, influential-case screening) could not be verified without access to the underlying case-level dataset; we flag this explicitly as a limitation of the regression model rather than presenting it as a fully diagnosed, confirmatory analysis, and we have retitled this subsection and softened the corresponding statements in the Discussion and Conclusion accordingly.

## Discussion

This study aimed to examine gut microbial composition, intestinal barrier function, and feeding behavior in the same children with ASD, rather than treating them as independent lines of evidence. There are three key findings. The results showed that children with ASD had significantly higher levels of zonulin and calprotectin in their stool compared with children who were neurotypical, confirming that both increased intestinal permeability and low-grade intestinal inflammation were prevalent in this group. Second, groups had significant differences in their gut bacterial composition, with ASD showing decreases in *Prevotella*, *Bifidobacterium*, and *Lactobacillus*, and increases in *Clostridium difficile* and *Bacteroidetes*. Third, and potentially most enlightening, was the lack of difference between the ASD and control groups in Total BAMBI scores, while within the ASD group, BAMBI scores more strongly correlated with some of the same taxa but with none of CARS, zonulin, or calprotectin.

The increased concentration of zonulin in feces is consistent with the concept of the “leaky gut” in ASD, where the permeability of the intestinal barrier allows lipopolysaccharide and other microbial molecules to enter the bloodstream and, possibly, trigger neuroinflammation [26]. The increased GI symptom burden previously reported in ASD is broadly in alignment with the higher calprotectin values observed here [27], although, as noted above, the ASD group’s mean calprotectin fell in the manufacturer’s borderline rather than elevated range; therefore, this should be read as suggestive of, rather than firm evidence for, an active mucosal inflammatory process in most children in this sample. Our data do not support such a linear pathway from permeability/inflammation to feeding behavior, at least in this sample, since there was little to no relation between the two outcomes (zonulin and calprotectin), and only a weak relationship between the permeability and feeding behaviors (BAMBI scores).

The microbial picture is more directly related to feeding behavior. The depletion of *Prevotella* and related saccharolytic genera in our ASD sample is in keeping with some of the first culture and sequencing-based findings in this area [28], as they have been shown to help maintain gut barrier and modulate local immune tone [29]. Similarly, both *Bifidobacterium* and *Lactobacillus*, both widely protective commensals, were depleted in the same manner, and the depletion of *Bifidobacterium* was the most strongly correlated with BAMBI severity in our correlation and regression analyses. The presence of *Clostridium difficile*—a recognized enteric pathogen rather than a neutral commensal—was both more frequently detected and more abundant in ASD, with abundance increasing with feeding-behavior severity [30]. Similarly, the literature on *Bacteroidetes* in ASD is genuinely mixed; in some samples, there is evidence of depletion, in others, enrichment [31]; therefore, we would not want to claim that this finding was consistently observed beyond our sample.

As noted in Section “Correlations between feeding/mealtime problem severity, autism severity, biomarkers, and microbiota”, after Bonferroni correction, the *Prevotella* and *Lactobacillus* correlations with BAMBI severity should be regarded as nominal/trend-level rather than confirmed associations; we retain the discussion of these taxa here because of their well-documented depletion in ASD in the wider literature, but our strongest, correction-robust claims in this study are limited to *Bifidobacterium, Clostridium difficile,* and *Bacteroidetes*.

Combined, these correlations indicate that feeding and mealtime issues in ASD might exist as a distinct aspect of the phenotype, one that is more closely related to the bacterial composition of the gut than to the severity of the ASD. This is in line with the findings of short courses of vancomycin, as well as microbiota transfer therapy that lasts for longer periods of time, on improvement of GI and behavioral symptoms without altering the child’s basic autism diagnosis and severity rating [18, 19]. It also provides a partial explanation for the lack of differences in Total BAMBI between our two groups: if feeding severity was more related to a child’s position on a microbial dysbiosis continuum than to the presence or absence of ASD, then a simple binary comparison of BAMBI scores, without comparing to microbiota, would likely underestimate the relationship.

### Strengths

The major advantage of this study was its multilevel design: all children had clinical severity assessed, feeding behaviors measured, two independent gut barrier markers, and six bacterial taxa quantified; this is because most of the previous studies on the gut microbiome in children with ASD have only compared children with ASD to healthy controls and therefore cannot conduct within-group correlation analyses. The case-control design was well matched on age, sex, and most socioeconomic and perinatal variables, minimizing the confounding of those variables. We also found that the use of both a detection/non-detection indicator and a quantitative Log_10_ CFU/g value for each taxon enabled us to differentiate between those taxa that were simply absent more frequently in ASD (*Prevotella*, *Bifidobacterium*, *Lactobacillus*) and those that were present with similar prevalence, but with a much greater difference in load when present (*Bacteroidetes*).

### Limitations

However, these findings are subject to some caveats. The cross-sectional design does not allow for causality to determine which is the cause and effect, e.g., a child’s limited diet may result from the pattern of microbes, or the pattern of microbes may result from the limited diet. The alpha and beta-diversity data from a broader survey of 16S rRNA amplicons were not available for this analysis. Last, the recruitment of participants in a single center in one part of Egypt restricts the generalizability of these findings to other groups and health-care settings.

Several additional limitations should be noted. First, diet and food selectivity, GI symptom severity (including constipation and reflux), and use of psychotropic medications, dietary supplements, probiotics, or behavioral interventions were not comprehensively measured in this study and could not be adjusted for; each of these factors can independently affect the gut microbiome, intestinal permeability, and mealtime behavior; therefore, residual confounding by these unmeasured factors cannot be excluded. Second, although the ASD and control groups were well matched on most demographic and perinatal variables, low birth weight was more frequent in the ASD group (Table 1); given that this was one of eleven baseline comparisons tested, it may in part reflect multiple testing rather than a true group difference, but as birth weight can independently influence both the developing microbiome and gut barrier function, we could not rule out or formally adjust for its contribution without the individual-level dataset. Third, the qPCR-based Log_10_ CFU-equivalent quantification used here is a semi-quantitative, non-culture-calibrated measure that was not normalized to total bacterial load, as detailed in the Methods; the low detection rates observed for several taxa also restricted the abundance comparisons in Table 3 to taxon-positive subsamples, which may not be fully representative of each group as a whole. Fourth, the BAMBI is a mealtime-behavior screening measure rather than a diagnostic tool for feeding or eating disorders, and no formal diagnostic assessment for such disorders was performed. Fifth, fecal zonulin is not yet a standardized clinical biomarker, and no a priori sample-size calculation was performed for this study (see Methods).

### Conclusions

The children with autism in this group showed indications of a compromised intestinal barrier, a low-grade inflammatory response in the gut, and marked microbial imbalances. On closer inspection, however, within the autism group, a very strong clinical picture arose. No correlation was found with the overall severity of their core developmental symptoms or systemic markers of leaky gut (zonulin and calprotectin) and the severity of their mealtime symptoms and food refusal.

Instead, these feeding problems were closely correlated with the composition of the bacteria residing within their digestive system.

If replicated in longitudinal and interventional studies, these bacterial associations could represent a candidate target for future gut-directed research (e.g., probiotic supplementation, dietary modification, or microbiota-based approaches) aimed at feeding and mealtime problems in ASD, potentially as a complement to, rather than a replacement for, established behavioral interventions. We emphasize that this cross-sectional, correlational study cannot establish that modifying the microbiome would improve feeding behavior, and any such approach would need to be tested in adequately powered, controlled clinical trials before being considered for clinical practice.

Longitudinal studies are required to determine if any of the associations found in this study are causal and repeatable, in addition to dietary intervention or probiotic supplementation, and full genome sequencing of the microbiome.

## Data Availability

The datasets generated and analyzed during the current study are available from the corresponding author on reasonable request.
